# Efficacy of sucrose and povidone–iodine mixtures in peritoneal dialysis catheter exit-site care

**DOI:** 10.1186/s12882-024-03591-1

**Published:** 2024-05-02

**Authors:** Takashin Nakayama, Kohkichi Morimoto, Kiyotaka Uchiyama, Naoki Washida, Ei Kusahana, Eriko Yoshida Hama, Ryunosuke Mitsuno, Shun Tonomura, Norifumi Yoshimoto, Akihito Hishikawa, Aika Hagiwara, Tatsuhiko Azegami, Jun Yoshino, Toshiaki Monkawa, Tadashi Yoshida, Shintaro Yamaguchi, Kaori Hayashi

**Affiliations:** 1 Division of Endocrinology, Metabolism and Nephrology, Department of Internal Medicine, Keio University School of Medicine, 35 Shinanomachi, Shinjuku-Ku, Tokyo, 160-8582 Japan; 2 Apheresis and Dialysis Center, Keio University School of Medicine, Tokyo, Japan; 3 Department of Nephrology, International University of Health and Welfare School of Medicine, Chiba, Japan; 4 Medical Education Center, Keio University School of Medicine, Tokyo, Japan

**Keywords:** Sucrose, Povidone–iodine, Peritoneal dialysis, Exit-site infections, Tunnel infections

## Abstract

**Background:**

Exit-site infection (ESI) is a common recurring complication in patients undergoing peritoneal dialysis (PD). Sucrose and povidone–iodine (SPI) mixtures, antimicrobial ointments that promote wound healing, have been used for the treatment of ulcers and burns, but their efficacy in exit–site care is still unclear.

**Methods:**

This single-center retrospective observational study included patients who underwent PD between May 2010 and June 2022 and presented with episodes of ESI. Patients were divided into SPI and non-SPI groups and followed up from initial ESI onset until PD cessation, death, transfer to another facility, or June 2023.

**Results:**

Among the 82 patients (mean age 62, [54–72] years), 23 were treated with SPI. The median follow-up duration was 39 months (range, 14–64), with an overall ESI incidence of 0.70 episodes per patient-year. Additionally, 43.1% of second and 25.6% of third ESI were caused by the same pathogen as the first. The log-rank test demonstrated significantly better second and third ESI-free survival in the SPI group than that in the non-SPI group (*p* < 0.01 and *p* < 0.01, respectively). In a Cox regression analysis, adjusting for potential confounders, SPI use was a significant predictor of decreased second and third ESI episodes (hazard ratio [HR], 0.22; 95% confidence interval [CI], 0.10–0.52 and HR, 0.22; 95%CI, 0.07–0.73, respectively).

**Conclusions:**

Our results showed that the use of SPI may be a promising option for preventing the incidence of ESI in patients with PD.

**Trial registration:**

This study was approved by the Keio University School of Medicine Ethics Committee (approval number 20231078) on August 28, 2023. Retrospectively registered.

**Supplementary Information:**

The online version contains supplementary material available at 10.1186/s12882-024-03591-1.

## Background

Exit-site infection (ESI) is one of the most common complications associated with peritoneal dialysis (PD). It can lead to tunnel infection (TI) and peritonitis, which together account for approximately 20% of reasons for PD discontinuation [1–3]. Although ESI typically responds to antimicrobial therapy, certain bacteria cause refractory infections, requiring surgical intervention [2–6]. Therefore, preventing the incidence of ESI is vital. However, to date, there is no consensus on how to provide daily exit-site care: the need for and type of topical disinfection remains controversial [7, 8].

A topical mixture of sucrose and povidone–iodine (SPI) combines the wound-healing and exudate-absorbing properties of sugar with the antimicrobial action of iodine [9, 10]. Its sugar component has been reported to inhibit biofilm formation by suppressing glycocalyx production [11, 12]. Since Knutson et al. reported in 1981 that SPI was useful for the treatment of multiple wound types, SPI has been widely used for treating diabetic ulcers, burns, and surgical wounds [13–17]. Currently, the Japanese Society of Pressure Ulcers recommends topical SPI use for pressure ulcers with inflammation and infection [18].

Skin injury caused by poor catheter immobilization or mechanical stress could provide an entry point for infectious organisms, leading to ESI [19, 20]. Some of the bacteria, such as *Staphylococcus aureus* and *Pseudomonas aeruginosa* form biofilms, making treatment difficult [1, 4, 21, 22]. In this context, SPI appears to be a reasonable option for managing PD catheter exit-site care. However, to our best knowledge, there are no reports on the efficacy of SPI in exit-site management. We therefore conducted a retrospective observational study including patients with PD and a history of ESI to investigate the efficacy of topical SPI.

## Methods

### Participants

The Keio University School of Medicine Ethics Committee reviewed and approved this study and its protocols (approval number, 20231078). Informed consent was obtained using an opt-out method available on our website. We included patients who underwent PD between May 2010 and June 2022 at our hospital. A history of ESI is a major risk factor for subsequent recurrence. Some patient populations experience repeat ESIs, whereas others never develop infections, with a lower likelihood of SPI administration [6, 19]. To reduce the variation in patients’ backgrounds and appropriately evaluate the effectiveness of SPI, we excluded patients without a history of ESI. Patients younger than 18 years of age were also excluded.

At our hospital, patients undergoing PD visit the outpatient clinic once a month (or more if necessary). Clinicians and nurses instructed patients to perform exit-site care daily, beginning with washing with soap and tap water after showering (more often if sweating profusely), followed by disinfection with povidone-iodine (10%) or chlorhexidine gluconate (0.5%), securely anchoring the tape, and covering with a sterile dressing. In addition, we recommended that patients avoid bathing, swimming, or marine sports that could entail risks of exposure of exit sites to possibly contaminated water. Neither antibiotic creams nor ointments were routinely applied to PD catheter exit sites for daily care. All patients underwent placement of double- or triple-cuffed, straight-tip, swan-neck silicone catheter with a lower-abdominal exit using the open surgical technique.

ESI was diagnosed based on the presence of purulent drainage from the exit site [7]. We determined the reappearance of pus within one month of improvement in clinical symptoms as the single ESI series. TI was defined as the presence of inflammation along the catheter–tunnel pathway. Peritonitis was diagnosed based on presence of abdominal pain and/or cloudy dialysis effluent, dialysis effluent white blood cell count > 100/cm^3^ with polynuclear leukocyte count > 50%, and positive dialysis effluent culture [8]. Peritonitis was considered to be associated with catheter-related infections if it occurred within two months after ESI onset or during treatment [1].

### Follow-up

The SPI, U–PASTA™ (70% sucrose and 3% povidone–iodine, Kowa, Nagoya, Japan), was used. The patients were classified into groups according to whether SPI was administered after the initial diagnosis of ESI. SPI was used not only during ESI, but also in the aberrant exit-site conditions including crust, swelling, erythema, granulation tissue, or exudative drainage (not the normal exit-site conditions). All participants were followed up from the initial onset of ESI until PD cessation (complete transition to hemodialysis [HD] or kidney transplantation), death, transfer to another facility, or study end (June 2023). The primary outcome was second and third ESI-free survival. Secondary outcomes were catheter infection-related surgical intervention-free survival (incisional drainage, unroofing, subcutaneous pathway diversion [SPD], or catheter removal) and peritonitis-free survival.

### Data collection

Demographic and anthropometric data at the time of ESI onset were collected from the patients’ electronic medical records. These data comprised age, sex, smoking history, housemate, body mass index (BMI; kg/m^2^), blood pressure (mmHg), concomitant TI, cause of end-stage renal disease, comorbidities, and PD vintage (months). Data on exit-site disinfectants (povidone-iodine [10%] versus chlorhexidine gluconate [0.5%]), type of catheters (double versus triple catheter cuffs), and dialysis modalities (use of an automated cycler, and combination with HD) were also collected. The Charlson comorbidity index (CCI) was calculated from these records. Additionally, we collected biochemical data: serum albumin (mg/dL), protein (mg/dL), creatinine (mg/dL), urea nitrogen (mg/dL), sodium (mEq/L), potassium (mEq/L), calcium (mg/dL), phosphorus (mg/dL), intact parathyroid hormone (pg/mL), iron (μg/dL), ferritin (ng/mL), and total iron-binding capacity (μg/dL). These biochemical data also included total cholesterol, β2-microglobulin (μg/mL), C-reactive protein (mg/dL), white blood cell count (10^3^/µL), hemoglobin (mg/dL), and platelet count (10^3^/µL). The geriatric nutritional risk index (GNRI) was calculated as 14.89 × serum albumin level (g/dL) + 41.7 × BMI/22 [23]. Information on the organisms cultured from exit-site swabs was also collected from the records.

### Statistical analyses

Continuous and binary variables are expressed as medians (25–75% interquartile range) and percentages, respectively. Normally and non-normally distributed continuous variables (using the Kolmogorov–Smirnov test) were compared between the groups using the unpaired Student’s *t*-test and Mann–Whitney U-test, respectively. Differences in binary variables between the groups were evaluated using Fisher’s exact test.

Survival curves were created using the Kaplan–Meier method. Differences in survival between the groups were compared using the log-rank test. Cox proportional models were used to determine hazard ratios (HRs) with 95% confidence intervals (CIs) for survival. In addition to SPI use, parameters that clinically appeared to be associated with ESI (age, sex, CCI, GNRI, concomitant TI, and use of systemic antibiotics) were fitted in the multivariate models as candidate independent variables. However, since a strong relationship between concomitant TI and the use of systemic antibiotics was found, considering the multicollinearity we adopted concomitant TI as a covariate. Since topical antibiotic use showed a significant difference between the groups, it was added as an independent variable. To simplify the analysis, the same independent variables were employed in the multivariate analyses to evaluate their association with catheter infection-related surgical interventions and peritonitis. As a sensitivity analysis of ESI recurrence, we performed multivariate analyses adjusting for diabetes mellitus instead of CCI. Furthermore, we excluded cases of treatment failure of the first ESI episode accompanied by surgical interventions and progression to peritonitis. Moreover, in addition to the cause-specific hazard model for standard Cox regression, we performed an analysis using the sub-distributional hazard model proposed by Fine and Gray, considering complete transition to HD and death as competing risk events [24].

All statistical analyses were performed using EZR (Saitama Medical Center, Jichi Medical University, Saitama, Japan), a graphical user interface for R (The R Foundation for Statistical Computing, Vienna, Austria) [25]. Statistical significance was defined as a two-sided *p*-value of < 0.05.

## Results

### Patient information

**Fig. 1:**
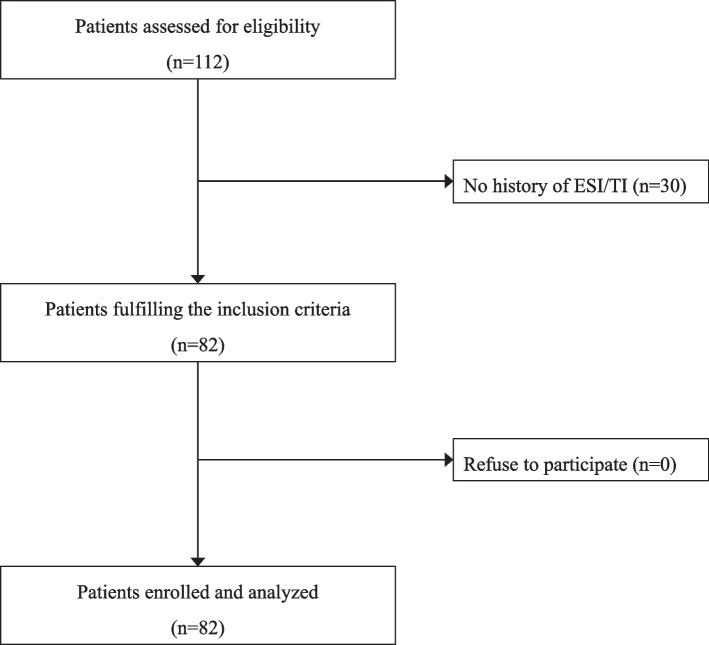
Patient flow chart

Of the eligible 112 patients who had initiated PD during the study period, 30 were excluded for having no history of ESI. Eventually, 82 patients were included (Fig. 1): 59 (72.0%) did not receive SPI and 23 (28.0%) received SPI after the first onset of ESI. The median follow-up period was 39 (14–64) months. Thirty-three (40.2%) patients were completely transitioned to HD, 13 (15.9%) died, four (4.9%) underwent kidney transplantation, and eight (9.8%) were transferred to other facilities.

Table 1 presents the baseline characteristics of the total study population, divided into SPI non-use or use. Overall, the median age and PD vintage were 62 (54–72) years and 11 (8–19) months, respectively. The female and diabetic mellitus proportions were 23.2% and 42.7%, respectively. In 15.9% of the patients, progression to TI had already occurred at the time of first ESI diagnosis. The SPI group was less likely to use topical antibiotics as the initial treatment of ESI compared with the non-SPI control group, likely because the SPI/antibiotic combination was cumbersome due to the nature of preparations (26.9 versus 67.8%, respectively; *p* < 0.01). There were no significant differences in the remaining variables between the groups, including the use of systemic antibiotics. Finally, no significant differences in biochemical data were observed between the groups (Table 2).

**Table 1:** Baseline characteristics at first ESI

Variables	Total (*N* = 82)	Non-SPI (*n* = 59)	SPI (*n* = 23)	*P* value
Age^a^ (year)	62 (54–72)	62 (55–72)	60 (50–70)	0.45
Sex (female)	19 (23.2)	11 (18.6)	8 (34.8)	0.15
Smoking history	48 (58.5)	36 (61.0)	12 (52.2)	0.62
Housemate	66 (80.5)	45 (76.3)	21 (91.3)	0.21
PD vintage^b^ (month)	11 (8–19)	10 (8–19)	13 (10–19)	0.27
BMI^a^	23.2 (21.5–25.7)	23.0 (21.1–24.9)	24.5 (22.7–27.8)	0.13
GNRI^a^	93.5 (87.2–102.3)	93.7 (87.2–99.7)	93.2 (88.0–104.9)	0.65
Mean blood pressure^a^ (mmHg)	98 (85–107)	98 (82–107)	99 (88–106)	0.36
Concomitant TI	13 (15.9)	8 (13.6)	5 (21.7)	0.50
Exit-site disinfectants (10% povidone-iodine/chlorhexidine)	76/6 (92.7/7.3)	56/3 (94.9/5.1)	20/3 (87.0/13.0)	0.34
Types of catheters (double/triple cuffs)	70/13(85.4/14.6)	51/8(86.4/13.6)	19/4(82.6/17.4)	0.73
Dialysis modality				
CAPD/APD	70/12 (85.4/14.6)	50/9 (84.7/15.3)	20/3 (87.0/13.0)	1.00
PD + HD combination	9 (11.0)	5 (8.5)	4 (17.4)	0.26
Underlying causes of ESRD				
Diabetic kidney disease	31 (37.8)	22 (37.3)	9 (39.1)	0.96
Nephrosclerosis	21 (25.6)	16 (27.1)	5 (21.7)	
Glomerulonephritis	18 (22.0)	13 (22.0)	5 (21.7)	
Others	12 (14.6)	8 (13.6)	4 (17.4)	
Comorbidities				
Diabetes mellitus	35 (42.7)	25 (42.4)	10 (43.5)	1.00
Cerebrovascular disease	10 (12.2)	9 (15.3)	1 (4.3)	0.27
Heart failure	29 (35.4)	20 (33.9)	9 (39.1)	0.80
Malignancy	8 (9.8)	6 (10.2)	2 ( 8.7)	1.00
Charlson comorbidity index^a^	4 (3–5)	4 (3–5)	4 (3–5)	0.96
Initial treatment for first ESI				
Topical antibiotics	46 (56.1)	40 (67.8)	6 (26.9)	< 0.01
Systemic antibiotics	45 (54.9)	31 (52.5)	14 (60.9)	0.62

*Abbreviations*: *ESI* Exit-site infection, *SPI* Sucrose and povidone-iodine, *PD* Peritoneal dialysis, *BMI* Body mass index, *GNRI* Geriatric nutritional risk index, *TI* Tunnel infection, *CAPD* Continuous ambulatory peritoneal dialysis, *APD* Automated peritoneal dialysis, *HD* Hemodialysis, *ESRD* End-stage renal disease, *RAS* Renin angiotensin system

^a^*P* value was obtained using the Unpaired T test

^b^*P* value was obtained using the Mann–Whitney U test

**Table 2:** Laboratory data at first ESI

Variables	Total (*N* = 82)	Non-SPI (*n* = 59)	SPI (*n* = 23)	*P* value
Albumin^a^ (mg/dL)	3.3 (3.0–3.6)	3.4 (3.0–3.7)	3.3 (3.0–3.5)	0.36
Total protein^a^ (mg/dL) (*n* = 81)	6.1 (5.8–6.5)	6.1 (5.8–6.5)	6.1 (5.8–6.5)	0.78
Creatinine^a^ (mg/dL)	10.6 (8.4–13.0)	10.5 (8.3–13.3)	11.4 (8.8–12.9)	1.00
Urea nitrogen^a^ (mg/dL)	57.7 (49.0–68.4)	57.4 (50.3–67.3)	58.0 (45.8–74.2)	0.85
White blood cells^b^ (10^3^μL)	6.5 (5.3–7.7)	6.4 (5.4–7.3)	7.1 (5.3–8.2)	0.29
Hemoglobin^b^ (g/dL)	10.4 (9.8–11.2)	10.4 (9.8–11.1)	10.7 (9.8–11.4)	0.95
Platelets^a^ (10^3^/μL)	211 (172–256)	208 (167–257)	212 (184–244)	0.89
Sodium^a^ (mEq/L)	138.2 (135.4–140.0)	138.0 (135.2–139.8)	139.2 (136.5–140.2)	0.26
Potassium^a^ (mEq/L)	4.6 (4.3–5.2)	4.5 (4.2–5.2)	4.6 (4.5–5.1)	0.99
Corrected calcium^b^ (mg/dL)	9.3 (8.9–9.7)	9.4 (8.9–9.8)	9.1 (8.8–9.6)	0.18
Phosphorus^a^ (mg/dL)	5.5 (4.8–6.1)	5.5 (4.9–6.0)	5.2 (4.7–6.6)	0.34
Intact PTH^b^ (pg/mL) (*n* = 78)	210 (127–283)	210 (127–281)	194 (132–325)	0.57
Iron^a^ (μg/dL) (*n* = 81)	81 (59–92)	81 (67–96)	78 (52–88)	0.17
Ferritin^b^ (ng/mL) (*n* = 79)	117 (68–193)	129 (73–193)	90 (59–164)	0.20
TIBC^a^ (μg/dL) (*n* = 81)	256 (232–278)	258 (236–278)	245 (224–280)	0.77
Total cholesterol^a^ (mg/dL) (*n* = 78)	161 (136–188)	160 (138–186)	172 (132–211)	0.32
β2-microglobulin^b^ (mg/L) (*n* = 74)	22.8 (17.4–29.3)	22.0 (17.4–26.9)	28.1 (18.2–33.8)	0.17
C-reactive protein^b^ (mg/L)	0.09 (0.03–0.23)	0.09 (0.03–0.19)	0.14 (0.04–0.38)	0.17

*Abbreviations*: *ESI* Exit site infection, *SPI* Sucrose and povidone–iodine, *PTH* Parathyroid hormone, *TIBC* Total iron binding capacity

^a^*P* value was obtained using the Unpaired T test

^b^*P* value was obtained using the Mann–Whitney U test

### ESI causative bacteria

Likely causative pathogens are listed in Table 3. The most common initial cause was *Staphylococcus aureus* (32.9%), followed by other gram-positive bacteria (31.7%), Coagulase-negative *Staphylococcus* (17.1%), other gram-negative bacteria (7.3%), *Pseudomonas aeruginosa* (6.1%), and *Mycobacteria* spp. (4.9%). No patients had negative cultures. Overall, gram-positive bacteria were mainly responsible for the initial ESI, a trend that was also observed in second and third episodes. We observed that 43.1% of the second and 25.6% of the third episodes were caused by the same organism detected at the first episode; 37.2% of the third episodes resulted from the same organisms found at the second episode.

**Table 3:** Distribution of ESI causative bacteria

Organisms	First ESI (*N* = 82)	Second ESI (*n* = 58)	Third ESI (*n* = 43)
Gram-positive			
*Staphylococcus aureus*	27 (32.9)	13 (22.4)	5 (11.6)
*Coagulase-negative staphylococcus*	14 (17.1)	13 (22.4)	7 (16.3)
Other gram-positive bacteria	26 (31.7)	20 (34.5)	17 (39.5)
Gram-negative			
*Pseudomonas aeruginosa*	5 (6.1)	4 (6.9)	2 (4.7)
Other gram-negative bacteria	6 (7.3)	3 (8.6)	5 (11.6)
Others			
*Candida* spp	0 (0.0)	0 (0.0)	3 (7.0)
*Mycobacteria* spp	4 (4.9)	2 (3.4)	3 (7.0)
Negative	0 (0.0)	3 (5.2)	1 (2.3)
Same species as the first time	–	25 (43.1)	11 (25.6)
Same species as the second time	–	–	16 (37.2)

*Abbreviations*: *ESI* Exit-site infection

### Association between SPI use and ESI

**Fig. 2:**
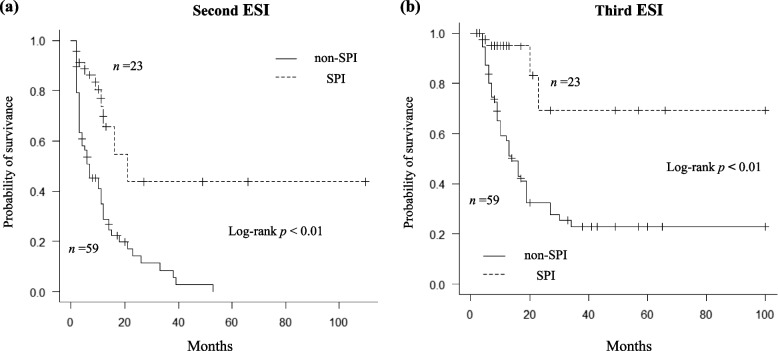
Kaplan–Meier analysis of (**a**) second and (**b**) third exit site infection-free survival time for groups divided according to non-use or use of SPI

We recorded 210 episodes of ESI over 301 patient-years, or 0.70 per patient-year, fifty-eight (70.7%) and 43 (52.4%) patients presented with a second and third ESI, respectively. The median second ESI-free time was significantly longer in the SPI compared with the non-SPI group (21 versus 7 months, *p* < 0.01) (Fig. 2a). Regarding the third development, the SPI group showed similarly favorable results (not reached versus 16 months, *p* < 0.01) (Fig. 2b). Using Cox proportional regression, SPI was independently associated with fewer second ESIs (HR, 0.22; 95%CI, 0.10–0.52); this model had a concordance index (C-index) of 0.65 (Table 4). Multivariate analyses to evaluate the risk of third ESI development also confirmed the significant effects of SPI (HR, 0.22; 95%CI, 0.07–0.73), with a C-index of 0.63. Using both sensitivity analyses, adjusting for diabetes mellitus (HR, 0.22; 95%CI, 0.10–0.52; HR, 0.20; 95%CI, 0.06–0.68, respectively) and excluding cases of treatment failure of the initial ESI episode (HR, 0.19; 95%CI, 0.08–0.49; HR, 0.22; 95%CI, 0.06–0.72, respectively), the significant association between SPI use and the subsequent risk of ESI persisted. The sub-distributional hazard models demonstrated that SPI use was significantly associated with the risk of subsequent ESI (HR, 0.23; 95%CI, 0.10–0.55 and HR, 0.20; 95%CI, 0.06–0.61, respectively) (Supplementary Table S3). Other variables were not significantly associated with ESI incidence using any of the multivariate models.

**Table 4:** Results of standard Cox proportional hazards for recurrence of ESI

Variables	Second		Third	
	HR (95%CI)	*P* value	HR (95%CI)	*P* value
Age (per 10 years)	1.01 (0.77–1.32)	0.95	1.12 (0.81–1.56)	0.49
Female (vs Male)	0.93 (0.47–1.86)	0.84	1.16 (0.53–2.53)	0.71
TI (vs non-TI)	0.51 (0.21–1.22)	0.13	0.54 (0.18–1.58)	0.26
CCI (per 1)	0.97 (0.80–1.19)	0.80	0.94 (0.75–1.19)	0.62
GNRI (per 10)	1.02 (0.73–1.43)	0.90	1.24 (0.87–1.78)	0.24
Use of SPI	0.22 (0.10–0.52)	< 0.01	0.22 (0.07–0.73)	0.01
Use of topical antibiotics	0.76 (0.40–1.44)	0.40	1.19 (0.58–2.45)	0.64

Concordance index for second and third ESI: 0.66 and 0.63, respectively

*Abbreviations*: *ESI* Exit-site infection, *HR* Hazard ratio, *CI* Confidence interval, *TI* Tunnel infection, *CCI* Charlson comorbidity index, *GNRI* Geriatric nutritional risk index, *SPI* Sucrose and povidone-iodine

### Association SPI use and catheter infection-related surgical interventions or peritonitis

**Fig. 3:**
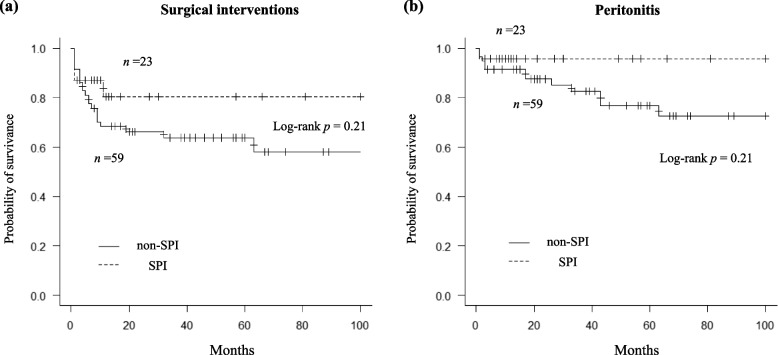
Kaplan–Meier analysis of catheter infection-related (**a**) surgical interventions and (**b**) peritonitis-free survival time for groups divided according to non-use or use of SPI

A total of 43 catheter infection-related surgical interventions were performed during the study period: 23 for catheter extraction, 10 for unroofing, 9 for SPD, and 1 for incisional drainage. Twenty-seven (32.9%) patients underwent at least one surgical intervention: 15 (18.3%), nine (11.0%), two (2.4%), and one (1.2%) underwent one, two, three, and four procedures, respectively. The median catheter infection-related surgical intervention-free time did not significantly differ between the groups (not reached versus 111 months, respectively; *p* = 0.21) (Fig. 3a). The standard Cox proportional hazard regression showed that SPI use did not significantly predict the risk of surgical intervention (HR, 0.79; 95%CI, 0.24–2.56), whereas female sex and concomitant TI were independently associated with its risk (HR, 0.21; 95%CI, 0.05–0.79 and HR, 22.75; 95%CI, 7.59–68.15, respectively) (Table 5). We recorded 16 episodes of catheter infection-related peritonitis (0.05 per patient-year; 0.20 per patient-year for all-cause peritonitis). Given the small number of events, the median times could not be calculated in either group (not reached versus not reached; *p* = 0.21) (Fig. 3b). Using the Cox proportional model, no variables, including SPI use (HR, 0.30; 95%CI, 0.04–2.48), were significantly associated with the risk of catheter infection-related peritonitis. However, concomitant TI had significant association (HR, 3.97; 95%CI, 1.01–15.56). The C-index for each standard hazard regression model was 0.85 and 0.69, respectively. The sub-distributional hazard model showed similar results (Supplementary Table S4).

**Table 5:** Results of standard Cox proportional hazards for catheter infection-related interventions and peritonitis

Variables	Surgical interventions		Peritonitis	
	HR (95%CI)	*P* value	HR (95%CI)	*P* value
Age (per 10 years)	1.18 (0.82–1.69)	0.37	0.94 (0.56–1.57)	0.81
Female (vs Male)	0.21 (0.05–0.79)	0.02	0.58 (0.12–2.87)	0.50
TI (vs non-TI)	22.75 (7.59–68.15)	< 0.01	3.97 (1.01–15.56)	0.05
CCI (per 1)	1.24 (0.91–1.68)	0.18	1.26 (0.84–1.89)	0.26
GNRI (per 10)	1.05 (0.62–1.76)	0.86	0.99 (0.48–2.03)	0.98
Use of SPI	0.79 (0.24–2.56)	0.69	0.30 (0.04–2.48)	0.26
Use of topical antibiotics	2.44 (0.87–6.80)	0.09	1.59 (0.41–6.27)	0.51

Concordance index for interventions and peritonitis: 0.85 and 0.69, respectively

*Abbreviations*: *HR* Hazard ratio, *CI* Confidence interval, *TI* Tunnel infection, *CCI* Charlson comorbidity index, *GNRI* Geriatric nutritional risk index, *SPI* Sucrose and povidone-iodine

## Discussion

Despite multiple advances in the management of PD, infections remain a crucial complication to be addressed and are a major predisposing factor for mortality, PD cessation, and hospitalization [1–3, 26, 27]. In particular, evidence on methods of PD catheter exit-site care is lacking. Therefore, we evaluated the effectiveness of SPI, which has attracted attention in other fields as an enhancer of ulcer and wound healing. Our multivariate model demonstrated that SPI use was associated with reduced risk of secondary ESI. However, its use did not show significant reductions in catheter infection-related surgical interventions or peritonitis. Concomitant TI at the first onset of ESI was a predictor. These findings indicated that SPI could be effective in preventing ESI, with early ESI diagnosis likely important for avoiding later invasive procedures and complications.

ESI incidence varies across time periods, facilities, and countries, generally ranging between 0.13 and 1.28 per patient-year [1, 6, 19, 28–30]. This wide variation may be partially attributed to the diverse definitions of ESI (such as presence of purulent drainage from the exit site or exit-site score ≥ 4) or diagnoses based on subjective observations [7, 20, 31]. The incidence of ESI in our study was higher (0.70 per patient-year), compared with the recent average rate in Japan (0.40 per patient-year) [32]. In contrast, we recorded an incidence of all-cause peritonitis of 0.20 per patient-year, near the Japanese average, achieving the target recommended by the International Society for Peritoneal Dialysis (ISPD) of 0.40 [7, 32]. Between 10 and 20% of all peritonitis cases have been estimated to be preceded by catheter infections, despite variations depending on the type of causative pathogen [33, 34]. As expected, the proportion of these patients was relatively high at 27.1%. Gram-positive bacteria were predominant in this study, consistent with findings in previous reports [5, 19, 33]. The details regarding the frequency discrepancy between peritonitis and ESI remain unclear. Nevertheless, the subjective reliance of ESI diagnosis on observational assessment may have had an impact. Additionally, the meticulous inspection of exit sites by physicians and nurses during each visit could have contributed to a higher probability of detecting ESI. Overall, the data on PD-related infections obtained from our hospital did not deviate significantly from the Japanese norms.

The ISPD guidelines recommend daily topical antibacterial treatments (although this recommendation has been downgraded according to the 2023 update), whereas the Japanese Society for Dialysis Therapy (JSDT) recommends the opposite opinion. We followed the recommendations by the JSDT in principle and therefore used these treatment less frequently [7, 8]. In our clinical setting, we confirmed that for exit sites in poor condition (assessed by the medical staff), physician-instructed SPI use prevented subsequent ESI. In those in good condition, SPI was not used because as it could take more handling time compared with conventional disinfectants. Sucrose, one SPI component, has been shown to enhance wound healing, sterility maintenance, and infection control [10, 12–14]. Although all forms of life require water for growth, sugars absorb exudates from their surroundings and mechanically clean necrotic tissue by creating an osmotic pressure difference [14, 35]. Sugars prevent and eliminate biofilms produced by a variety of organisms and function as a modulator of fibroblasts and keratinocytes [10, 12]. Povidone-iodine, the other component of SPI, is a well-known disinfectant with broad-spectrum antibiotic activity. However, this povidone-iodine component is cytotoxic and decreases fibroblast and macrophage survival, which may delay wound healing and promote secondary skin injury [36]. The iodine concentration in SPI is 3%, lower compared with more commonly used preparations (10%). Since its toxicity is concentration-dependent, SPI, with a low concentration of iodine and slow-release properties, is considered less harmful [37, 38]. Shiraishi et al. demonstrated that SPI with a low iodine concentration had sufficient antibiotic activity, despite taking slightly longer to sterilize compared with conventional povidone-iodine solutions [39]. The above SPI characteristics appear to favor its application to PD catheter exit-site care.

The therapeutic effect of SPI on existing ESI was also evaluated; unlike its preventive effects, no obvious beneficial effects were observed. However, we could not exclude the possibility that the small number of treatment efficacy-related outcomes made it difficult to appropriately evaluate these effects. The SPI group survival was not inferior to that in the non-SPI group. Therefore SPI use might have a positive impact. Given that SPIs have a lower risk of antimicrobial resistance and microbial substitution than do topical antibiotics, their use appears to be an effective, low-risk therapeutic option [40, 41].

Few studies have explored the risk factors for ESI. Based on the limited evidence available, ESI history, poor compliance with exit-site care, and mechanical injuries have been strongly associated with ESI occurrence [19, 20]. Moreover, there are no consistent findings regarding other factors, including age, sex, dialysis modality, or comorbidities [6, 19, 20, 30, 42]. We found no significant association between ESI development and any factor other than SPI use. Very few studies have directly evaluated subsequent outcomes of ESI. Au et al. reported that in patients with ESI caused by *Serratia* species, the presence of TI increases the risk of subsequent troublesome clinical courses [43]. In the present study, concomitant TI predicted events of surgical intervention and subsequent peritonitis regardless of the type of bacterium. These findings highlight the importance of early detection before progression of ESI to TI.

This study has several limitations. First, since this was a retrospective observational study, we were unable to adjust for some potential confounding factors, including a history of catheter pulling and mechanical stress, potentially leading to the modest predictive accuracy of all Cox models. As a matter of course, the direct causality between SPI use and low frequency of ESI development is also unclear. The patients with high presumably adherence to exit-site care might have been prioritized to receive SPI, taking time for management. Second, there are concerns regarding selection bias arising from the single-center design. Due to the limited sample size, the impact of SPI on the types of causative pathogens could not be evaluated. Also, only patients with a history of ESI were included in this study. This might limit the generalizability of our findings. However, the assessment of ESI relies on subjective rather than objective observations [20]. In terms of diagnostic consistency, this single-center design may have some merit. Furthermore, the situation surrounding PD-related infections was consistent with previous reports. Third, the patients did not routinely receive topical antibacterial therapy in the present study (in accordance with the recommendation by the JSDT, not the ISPD), the results of which should be interpreted with caution. However, it is noteworthy that although the SPI group used less local antimicrobial therapy, it had showed beneficial outcomes. Fourth, over the extensive 10-year observation period, a multitude of medical staff has been involved in the care for patients undergoing PD. Although accurate assessment is challenging, there is a possibility that the discretion in the use of SPI may not have been consistent. Finally, we compared the effectiveness of SPI with that of a disinfectant (mainly, povidone-iodine [10%]) under poor exit-site conditions. Our results may reflect the shortcomings of high-concentration povidone-iodine, such as local irritability. Indeed, several guidelines do not actively recommend its use [7, 8]. However, because this agent remains one of the most popular antiseptic materials for exit-site care in the real world, we suggest that the results of this study may have implications in daily clinical practice [44].

In conclusion, our study demonstrated that SPI use could be associated with a low risk of subsequent ESI in patients with PD and a history of ESI. Further definitive randomized controlled studies are required to confirm the efficacy of SPI.

### Supplementary Information

**Additional file 1:** **Table S1.** Results of Standard Cox proportional hazards for recurrence of ESI (adjusting for diabetes mellitus). **Table S2.** Results of Standard Cox proportional hazards for recurrence of ESI (excluding cases with treatment failure for the first ESI episode). **Table S3.** Results of Sub-distribution Cox proportional hazards for recurrence of ESI. **Table S4.** Results of Sub-distribution Cox proportional hazards for catheter infection-related interventions and peritonitis.

## Data Availability

The data supporting the findings of this study are available under reasonable request to the corresponding author.

## Abbreviations

ESI: Exit-site infection
PD: Peritoneal dialysis
TI: Tunnel infection
SPI: Sucrose and povidone–iodine
HD: Hemodialysis
SPD: Subcutaneous pathway diversion
CCI: Charlson comorbidity index

## References

[1] van Diepen AT, Tomlinson GA, Jassal SV (2012). The association between exit site infection and subsequent peritonitis among peritoneal dialysis patients. Clin J Am Soc Nephrol.

[2] Lloyd A, Tangri N, Shafer LA, Rigatto C, Perl J, Komenda P (2013). The risk of peritonitis after an exit site infection: a time-matched, case-control study. Nephrol Dial Transplant.

[3] Nakayama M, Miyazaki M, Honda K, Kasai K, Tomo T, Nakamoto H (2014). Encapsulating peritoneal sclerosis in the era of a multi-disciplinary approach based on biocompatible solutions: the next-Pd study. Perit Dial Int.

[4] Bunke M, Brier ME, Golper TA (1995). Pseudomonas peritonitis in peritoneal dialysis patients: the Network #9 Peritonitis Study. Am J Kidney Dis.

[5] Mujais S (2006). Microbiology and outcomes of peritonitis in North America. Kidney Int Suppl.

[6] Beckwith H, Clemenger M, McGrory J, Hisole N, Chelapurath T, Newbury S (2019). Repeat peritoneal dialysis exit-site infection: definition and outcomes. Perit Dial Int.

[7] Chow KM, Li PK, Cho Y, Abu-Alfa A, Bavanandan S, Brown EA (2023). ISPD catheter-related infection recommendations: 2023 update. Perit Dial Int.

[8] Ito Y, Ryuzaki M, Sugiyama H, Tomo T, Yamashita AC, Ishikawa Y (2021). Peritoneal dialysis guidelines 2019 Part 1 (Position paper of the Japanese Society for Dialysis Therapy). Ren Replace Ther.

[9] Zamora JL (1986). Chemical and microbiologic characteristics and toxicity of povidone-iodine solutions. Am J Surg.

[10] Nakao H, Yamazaki M, Tsuboi R, Ogawa H (2006). Mixture of sugar and povidone–iodine stimulates wound healing by activating keratinocytes and fibroblast functions. Arch Dermatol Res.

[11] Yasuhiro N (2013). External use iodine preparations for pressure ulcers and chronic skin ulcers. Jpn J Pharm Palliat Care Sci.

[12] Lu J, Cokcetin NN, Burke CM, Turnbull L, Liu M, Carter DA (2019). Honey can inhibit and eliminate biofilms produced by Pseudomonas aeruginosa. Sci Rep.

[13] Knutson RA, Merbitz LA, Creekmore MA, Snipes HG (1981). Use of sugar and povidone-iodine to enhance wound healing: five year's experience. South Med J.

[14] Chirife J, Scarmato G, Herszage L (1982). Scientific basis for use of granulated sugar in treatment of infected wounds. Lancet.

[15] Anania WC, Rosen RC, Wallace JA, Weinblatt MA, Gerland JS, Castillo J (1985). Treatment of diabetic skin ulcerations with povidone–iodine and sugar. Two case reports. J Am Podiatr Med Assoc.

[16] Topham J (1996). Sugar paste and povidone–iodine in the treatment of wounds. J Wound Care.

[17] Di Stadio A, Gambacorta V, Cristi MC, Ralli M, Pindozzi S, Tassi L (2019). The use of povidone-iodine and sugar solution in surgical wound dehiscence in the head and neck following radio-chemotherapy. Int Wound J.

[18] Japanese Society of Pressure Ulcers (2005). Guideline for Local Treatment of Pressure Ulcers.

[19] Lin J, Ye H, Li J, Qiu Y, Wu H, Yi C (2020). Prevalence and risk factors of exit-site infection in incident peritoneal dialysis patients. Perit Dial Int.

[20] Ding XR, Huang HE, Liao YM, Zhu JR, Tang W, Fang XW (2021). Daily self-care practices influence exit-site condition in patients having peritoneal dialysis: a multicenter cross-sectional survey. J Adv Nurs.

[21] Racenis K, Kroica J, Rezevska D, Avotins L, Skuditis E, Popova A (2020). S. aureus colonization, biofilm production, and phage susceptibility in peritoneal dialysis patients. Antibiotics (Basel).

[22] Khan W, Bernier SP, Kuchma SL, Hammond JH, Hasan F, O'Toole GA (2010). Aminoglycoside resistance of Pseudomonas aeruginosa biofilms modulated by extracellular polysaccharide. Int Microbiol.

[23] Minamisawa M, Miura T, Motoki H, Ueki Y, Nishimura H, Shimizu K (2018). Geriatric nutritional risk index predicts cardiovascular events in patients at risk for heart failure. Circ J.

[24] Fine JP, Gray RJ (1999). A proportional hazards model for the subdistribution of a competing risk. J Am Stat Assoc.

[25] Kanda Y (2013). Investigation of the freely available easy-to-use software 'EZR' for medical statistics. Bone Marrow Transplant.

[26] Jaar BG, Plantinga LC, Crews DC, Fink NE, Hebah N, Coresh J (2009). Timing, causes, predictors and prognosis of switching from peritoneal dialysis to hemodialysis: a prospective study. BMC Nephrol.

[27] Choi P, Nemati E, Banerjee A, Preston E, Levy J, Brown E (2004). Peritoneal dialysis catheter removal for acute peritonitis: a retrospective analysis of factors associated with catheter removal and prolonged postoperative hospitalization. Am J Kidney Dis.

[28] Scalamogna A, Castelnovo C, De Vecchi A, Ponticelli C (1991). Exit-site and tunnel infections in continuous ambulatory peritoneal dialysis patients. Am J Kidney Dis.

[29] Bernardini J, Bender F, Florio T, Sloand J, Palmmontalbano L, Fried L (2005). Randomized, double-blind trial of antibiotic exit site cream for prevention of exit site infection in peritoneal dialysis patients. J Am Soc Nephrol.

[30] Eriguchi M, Tsuruya K, Yoshida H, Haruyama N, Tanaka S, Tsuchimoto A (2016). Extended swan-neck catheter with upper abdominal exit-site reduces peritoneal dialysis-related infections. Ther Apher Dial.

[31] Eriguchi M, Tsuruya K, Yoshida H, Yamada S, Tanaka S, Suehiro T (2011). Validation of the exit-site scoring system recommended by the 2005 guidelines of the International Society for peritoneal dialysis. Perit Dial Int.

[32] Masakane I, Taniguchi M, Nakai S, Tsuchida K, Goto S, Wada A (2018). Annual dialysis data report 2015, JSDT renal data registry. Ren Replace Ther.

[33] Sachar M, Shah A (2022). Epidemiology, management, and prevention of exit site infections in peritoneal dialysis patients. Ther Apher Dial.

[34] Brook NR, White SA, Waller JR, Nicholson ML (2004). The surgical management of peritoneal dialysis catheters. Ann R Coll Surg Engl.

[35] Gordon H, Middleton K, Seal K, Sullens K (1985). Sugar and wound healing. Lancet.

[36] Balin AK, Pratt L (2002). Dilute povidone-iodine solutions inhibit human skin fibroblast growth. Dermatol Surg.

[37] Lineaweaver W, Howard R, Soucy D, McMorris S, Freeman J, Crain C (1985). Topical antimicrobial toxicity. Arch Surg.

[38] Van den Broek PJ, Buys LF, Van Furth R (1982). Interaction of povidone-iodine compounds, phagocytic cells, and microorganisms. Antimicrob Agents Chemother.

[39] Shiraishi T, Takahashi N, Nakagawa Y (1992). Antibacterial activity of U-PASTA to MRSA and P. aeruginosa. PN Pharmacot Ther.

[40] Merckoll P, Jonassen TØ, Vad ME, Jeansson SL, Melby KK (2009). Bacteria, biofilm and honey: a study of the effects of honey on ‘planktonic’ and biofilm-embedded chronic wound bacteria. Scand J Infect Dis.

[41] Akira O (1996). Clinical and Bacteriological Research of sucrose/povidone-Iodine Ointment (U-PASTA kowa) for Pressure Sores and Skin Ulcers. Skin Res.

[42] Nessim SJ, Komenda P, Rigatto C, Verrelli M, Sood MM (2013). Frequency and microbiology of peritonitis and exit-site infection among obese peritoneal dialysis patients. Perit Dial Int.

[43] Au CWH, Yap DYH, Chan JFW, Yip TPS, Chan TM (2021). Exit site infection and peritonitis due to Serratia species in patients receiving peritoneal dialysis: epidemiology and clinical outcomes. Nephrology (Carlton).

[44] Young DJ (2014). Chronic exit-site care using povidone–iodine versus normal saline in peritoneal dialysis patients. Kidney Res Clin Pract.

