# Supplementary material for: Efficacy of sucrose and povidone–iodine mixtures in peritoneal dialysis catheter exit-site care
Source: BMC Nephrol. 2024 May 2;25:151. doi: 10.1186/s12882-024-03591-1 (PMC11064401; doi:10.1186/s12882-024-03591-1)
Supplement: Supplementary file 1 — Additional file 1: Table S1. Results of Standard Cox proportional hazards for recurrence of ESI (adjusting for diabetes mellitus). Table S2. Results of Standard Cox proportional hazards for recurrence of ESI (excluding cases with treatment failure for the first ESI episode). Table S3. Results of Sub-distribution Cox proportional hazards for recurrence of ESI. Table S4. Results of Sub-distribution Cox proportional hazards for catheter infection-related interventions and peritonitis. [file 12882_2024_3591_MOESM1_ESM.docx]

**Table S1. Results of Standard Cox proportional hazards for ESI recurrence (adjusting for diabetes mellitus)**

| Variables | Second | |  | Third | |
| --- | --- | --- | --- | --- | --- |
|  | HR (95%CI) | *P* value |  | HR (95%CI) | *P* value |
| Age (per 10 years) | 1.00 (0.77–1.28) | 0.97 |  | 1.14 (0.82–1.57) | 0.43 |
| Female (versus Male) | 0.94 (0.47–1.89) | 0.86 |  | 1.31 (0.61–2.82) | 0.49 |
| TI (versus non-TI) | 0.51 (0.21–1.24) | 0.14 |  | 0.46 (0.15–1.39) | 0.17 |
| Diabetes mellitus | 0.98 (0.55–1.73) | 0.95 |  | 0.94 (0.76–1.17) | 0.57 |
| GNRI (per 10) | 1.03 (0.73–1.43) | 0.89 |  | 1.28 (0.89–1.85) | 0.18 |
| Use of SPI | 0.22 (0.10–0.52) | < 0.01 |  | 0.20 (0.06–0.68) | < 0.01 |
| Use of topical antibiotics | 0.75 (0.40–1.41) | 0.37 |  | 1.08 (0.52–2.23) | 0.83 |
| Abbreviations: ESI, exit-site infection; HR, hazard ratio; CI, confidence interval; TI, tunnel infection; GNRI, geriatric nutritional risk index; SPI, sucrose and povidone-iodine. | | | | | |

**Table S2. Results of Standard Cox proportional hazards for ESI recurrence (excluding cases of treatment failure of the first ESI episode)**

| Variables | Second | |  | Third | |
| --- | --- | --- | --- | --- | --- |
|  | HR (95%CI) | *P* value |  | HR (95%CI) | *P* value |
| Age (per 10 years) | 0.99 (0.76–1.29) | 0.94 |  | 1.15 (0.84–1.58) | 0.38 |
| Female (versus Male) | 1.03 (0.51–2.07) | 0.94 |  | 1.32 (0.62–2.83) | 0.47 |
| TI (versus non-TI) | 1.32 (0.39–4.44) | 0.66 |  | 1.48 (0.43–5.14) | 0.53 |
| CCI (per 1) | 1.01 (0.81–1.26) | 0.91 |  | 1.01 (0.80–1.28) | 0.92 |
| GNRI (per 10) | 1.09 (0.77–1.55) | 0.61 |  | 1.28 (0.89–1.83) | 0.18 |
| Use of SPI | 0.19 (0.08–0.49) | < 0.01 |  | 0.22 (0.06–0.72) | 0.01 |
| Use of topical antibiotics | 0.64 (0.32–1.26) | 0.20 |  | 0.94 (0.45–1.93) | 0.86 |
| Abbreviations: ESI, exit-site infection; HR, hazard ratio; CI, confidence interval; TI, tunnel infection; CCI, Charlson comorbidity index; GNRI, geriatric nutritional risk index; SPI, sucrose and povidone-iodine. 71 patients were included in the analysis. | | | | | |

**Table S3. Results of sub-distribution Cox proportional hazards for ESI recurrence**

| Variables | Second | |  | Third | |
| --- | --- | --- | --- | --- | --- |
|  | HR (95%CI) | *P* value |  | HR (95%CI) | *P* value |
| Age (per 10 years) | 1.00 (0.77–1.28) | 0.97 |  | 1.11 (0.81–1.53) | 0.50 |
| Female (versus Male) | 1.01 (0.60–1.70) | 0.98 |  | 1.23 (0.62–2.46) | 0.55 |
| TI (versus non-TI) | 0.70 (0.35–1.40) | 0.31 |  | 0.57 (0.21–1.56) | 0.27 |
| CCI (per 1) | 0.99 (0.84–1.17) | 0.92 |  | 0.94 (0.76–1.17) | 0.57 |
| GNRI (per 10) | 0.99 (0.75–1.32) | 0.96 |  | 1.23 (0.84–1.79) | 0.29 |
| Use of SPI | 0.23 (0.10–0.55) | < 0.01 |  | 0.20 (0.06–0.61) | < 0.01 |
| Use of topical antibiotics | 0.91 (0.55–1.52) | 0.72 |  | 1.21 (0.62–2.35) | 0.57 |
| Abbreviations: ESI, exit site infection; HR, hazard ratio; CI, confidence interval; TI, tunnel infection; CCI, Charlson comorbidity index; GNRI, geriatric nutritional risk index; SPI, sugar and povidone-iodine. | | | | | |

**Table S4. Results of sub-distribution Cox proportional hazards for catheter infection-related interventions and peritonitis**

| Variables | Surgical interventions | |  | Peritonitis | |
| --- | --- | --- | --- | --- | --- |
|  | HR (95%CI) | *P* value |  | HR (95%CI) | *P* value |
| Age (per 10 years) | 1.14 (0.8–1.57) | 0.42 |  | 0.89 (0.57–1.41) | 0.62 |
| Female (versus Male) | 0.20 (0.07–0.61) | < 0.01 |  | 0.54 (0.17–1.66) | 0.28 |
| TI (versus non-TI) | 21.04 (8.04–55.06) | < 0.01 |  | 4.27 (0.89–20.47) | 0.07 |
| CCI (per 1) | 1.20 (0.90–1.60) | 0.21 |  | 1.18 (0.83–1.66) | 0.35 |
| GNRI (per 10) | 1.05 (0.67–1.64) | 0.84 |  | 0.93 (0.38–2.30) | 0.88 |
| Use of SPI | 0.78 (0.32–1.88) | 0.57 |  | 0.28 (0.04–2.04) | 0.21 |
| Use of topical antibiotics | 2.47 (1.14–5.35) | 0.02 |  | 1.67 (0.46–6.03) | 0.43 |
| Abbreviations: HR, hazard ratio; CI, confidence interval; TI, tunnel infection; CCI, Charlson comorbidity index; GNRI, geriatric nutritional risk index; SPI, sucrose and povidone-iodine. | | | | | |
